# Colorectal cancer liver metastasis: immunosuppressive microenvironment, signaling pathways, and emerging therapeutic strategies

**DOI:** 10.3389/fimmu.2026.1855761

**Published:** 2026-06-10

**Authors:** Hanlin Yang, Shaoxian Wu, Lujun Chen

**Affiliations:** 1 Department of Tumor Biological Treatment, The Third Affiliated Hospital of Soochow University, Changzhou, China; 2 Jiangsu Engineering Research Center for Tumor Immunotherapy, The Third Affiliated Hospital of Soochow University, Changzhou, China; 3 Institute for Cell Therapy of Soochow University, The Third Affiliated Hospital of Soochow University, Changzhou, China

**Keywords:** CD8+T exhaustion, colorectal cancer liver metastasis, immune checkpoint inhibitors, immunosuppressive microenvironment, signaling pathway

## Abstract

Colorectal cancer liver metastasis (CRLM) remains a leading cause of mortality in colorectal cancer. The metastatic liver site is characterized by an immunosuppressive microenvironment enriched with inhibitory immune cells and cytokines, contributing to a poorly inflamed, “cold” tumor phenotype. The progression of CRLM is driven by aberrant activation of oncogenic signaling pathways and a dysfunctional immune landscape, marked by T cell exhaustion and the expansion of immunosuppressive cell populations. This review summarizes the epidemiological characteristics of CRLM and discusses the mechanisms underlying its immunosuppressive microenvironment. We further highlight recent advances in therapeutic strategies, including radiotherapy, interventional approaches, and immune checkpoint inhibitors, providing insights for future mechanistic research and therapeutic development.

## 1 Introduction

Colorectal cancer (CRC) is among the most prevalent malignancies globally. Over recent decades, its incidence has continued to increase, with markedly higher rates in developed countries compared with developing regions. Despite advances in diagnosis and treatment, the mortality associated with CRC remains high (1). Challenges in early diagnosis, limited therapeutic options, and a high propensity for regional metastasis collectively contribute to the poor clinical prognosis of patients with CRC (2). The liver represents one of the most common sites of distant metastasis in CRC (3). This pattern is closely related to the anatomical features of the portal venous system, through which venous blood from the colorectum drains directly into the liver, facilitating hematogenous dissemination and hepatic colonization of tumor cells. As a result, the colorectal cancer liver metastasis (CRLM) has become a major determinant of patient prognosis and a leading cause of mortality (4). Consistent with this metastatic route, epidemiological data demonstrate a substantial incidence of CRLM. Approximately, 20%-30% of CRC patients develop liver metastases in the disease course, with most cases arising within three years following initial diagnosis, indicating a peak period of recurrence risk (2, 5). Synchronous metastases occur in approximately 15%-25% of patients and show a positive association with TNM stage (6, 7). In patients undergoing resection with curative intent, metachronous liver metastases predominantly emerge in the early postoperative phase, with cumulative incidences of 4%, 12%, and 16% at 1, 3, and 5 years, respectively, and a median interval of 17–20 months (7–9). Primary tumor location and patient characteristics also influence prognosis. This effect is stage-dependent: right-sided CRC is associated with worse survival in stage III–IV disease, but shows a relative advantage in stage II, possibly due to a higher prevalence of microsatellite instability (MSI). In addition, men have a higher incidence of liver metastasis than women at similar ages, suggesting that sex and age may affect metastatic patterns and outcomes (10, 11). Surgical resection combined with chemotherapy is considered the standard treatment for selected patients with CRLM. However, population-based studies have shown that only a subset of patients are eligible for curative-intent resection due to factors such as tumor burden, unfavorable anatomical location, extrahepatic disease, or patient-related conditions, and these patients generally experience significantly poorer survival outcomes compared with those undergoing resection (2, 7, 9). In clinical practice, approximately one quarter of patients present with liver metastases at the time of initial diagnosis. The presence or absence of hepatic involvement plays a pivotal role in determining overall survival (OS). Patients with localized disease often achieve relatively favorable long-term outcomes following appropriate treatment. However, once liver metastasis develops, prognosis deteriorates markedly and survival is substantially shortened (12). The emergence and effective management of liver metastases have become critical determinants in tailoring therapeutic approaches and evaluating prognosis in patients with CRC (13).

Notably, the liver is characterized by a unique immunosuppressive microenvironment that favors immune tolerance (14–16). This environment facilitates T cell exhaustion and promotes the secretion of multiple immunosuppressive mediators, such as interleukin-10 (IL-10) and transforming growth factor-β (TGF-β), by antigen-presenting cells (APCs) and Kupffer cells, thereby creating conditions that support metastatic colonization and immune evasion (14, 17). Consistent with these biological features, emerging clinical evidence from immune checkpoint inhibitor (ICI)-based therapies has demonstrated that the presence of liver metastases is associated with reduced therapeutic responses and inferior survival outcomes, highlighting the liver as a key site of immune resistance (18, 19). The tumor microenvironment (TME) of CRLM features multiple immune cell subsets, such as macrophages, T cells, and B cells, together with various cytokines and chemokines (20). In this context, CRLM commonly exhibit an immunologically “cold” phenotype. Metastatic lesions are characterized by prominent infiltration of regulatory T cells (Tregs) and M2-polarized tumor-associated macrophages (TAMs), accompanied by diminished cytotoxic activity of CD8^+^T cells. At the same time, the persistent presence of inhibitory cytokines and chemokines sustains and amplifies an immunosuppressive TME (21).

In recent years, the integration of systemic therapy with liver-directed approaches has substantially advanced the management of CRLM. On the systemic side, combination regimens incorporating fluoropyrimidine-based chemotherapy doublets (e.g., FOLFOX or FOLFIRI) combined with oxaliplatin or irinotecan, together with biologic agents such as bevacizumab or anti-EGFR monoclonal antibodies (cetuximab or panitumumab), and, in selected cases, immunotherapy, have improved tumor burden control and increased conversion-to-resection rates, ultimately contributing to better clinical outcomes (22, 23). Recent clinical advances in combination strategies, including ICIs combined with targeted therapies, have further demonstrated the potential to overcome resistance mechanisms associated with metastatic sites such as the liver (24). Meanwhile, liver-directed interventions–such as hepatic arterial infusion chemotherapy, radiofrequency ablation, and selective internal radiation therapy–have emerged as important complementary or alternative options. These strategies provide additional therapeutic opportunities, particularly for patients with initially un-resectable disease (25, 26). This review summarizes the epidemiological features and prognostic significance of CRLM, with a focus on the molecular mechanisms and immune remodeling processes that underlie its development. We further outline the key signaling pathways and microenvironmental factors involved in metastatic progression. Compared with previous reviews, we place particular emphasis on the interplay between oncogenic signaling pathways and the immunosuppressive microenvironment, especially in the context of metastatic progression and therapeutic resistance. Based on these insights, we discuss the biological basis of current therapeutic strategies and highlight potential molecular targets and mechanism-driven approaches, with the aim of informing precision treatment of CRLM.

## 2 Three major signaling pathways involved in CRLM

As both a key immunological organ and a central hub of systemic metabolism, the liver possesses a unique microenvironment that is highly permissive to tumor colonization. As a result, the liver is a common site of secondary spread for multiple primary tumors, such as CRC, melanoma, and breast cancer (20). CRLM is a highly intricate process in which only a small proportion of tumor cells successfully undergo epithelial–mesenchymal transition (EMT), traverse the extracellular matrix (ECM), infiltrate adjacent tissues, enter and withstand the circulation, and ultimately exit the bloodstream to establish metastatic growth at distant sites (27). CRLM progression is strongly driven by abnormal activation of several core oncogenic signaling networks, including the Wnt/β-catenin, RAS/RAF/MEK/ERK, and PI3K/AKT/mTOR pathways. Aberrant activity within these signaling networks may promote malignant cell expansion and simultaneously facilitates the establishment of an immunosuppressive landscape in the liver TME (28).

### 2.1 The canonical Wnt/β-catenin signaling pathway

The canonical Wnt/β-catenin signaling cascade is frequently upregulated across a wide range of cancers, particularly in CRC, where it may contribute to tumor cell proliferation while inhibiting apoptosis (29). In CRC, aberrant activation of the Wnt/β-catenin pathway may be driven by loss-of-function mutations in the *APC* gene, a key component of the β-catenin destruction complex. Disruption of *APC* leads to impaired degradation and subsequent nuclear accumulation of β-catenin, resulting in constitutive transcriptional activation of Wnt target genes and representing a fundamental initiating event in colorectal tumorigenesis (30, 31).

Through these effects, sustained Wnt/β-catenin signaling supports the maintenance of an immunosuppressive TME (32). In highly activated CRLM lesions, activation of this signaling axis correlates with diminished major histocompatibility complex class I (MHC class I) expression on malignant cells, thereby weakening their recognition and subsequent activation of CD8^+^ cytotoxic T cells. In parallel, increased levels of chemokines such as CCL22 and CXCL12 facilitate the recruitment of Tregs, a subset of CD4^+^T cells with potent immunosuppressive functions that limit effector T cell activation, and promote their accumulation within hepatic metastatic lesions (33, 34). Altogether, these changes are associated with an immunosuppressive TME and foster the emergence of an immunologically “cold” tumor phenotype. In CRLM cases exhibiting low WNT/β-catenin activity, substantial infiltration by CD8^+^T cells and NKs supports ongoing antitumor immune responses, while reduced numbers of Tregs and M2-polarized TAMs partially alleviate local immunosuppression (35, 36). Concurrent downregulation of immune checkpoints, including programmed death-ligand 1(PD-L1) and CTLA-4, has been implicated in improved responses to immune checkpoint inhibitors (ICIs) and significantly prolonged progression-free and OS (37, 38).

### 2.2 *KRAS*-driven remodeling of the immunosuppressive TME

*KRAS* mutations are present in approximately 40-50% of patients with CRLM and are closely associated with adverse clinical outcomes, including reduced OS and increased recurrence risk (39). Notably, distinct *KRAS* variants exhibit differential biological and clinical behaviors. For example, *KRAS* G12D and G12V mutations are among the most prevalent subtypes in CRC and are generally associated with more aggressive tumor phenotypes, enhanced metastatic potential, and poorer prognosis. In contrast, *KRAS* G12C, although less frequent in CRC, has emerged as a therapeutically targetable variant, with specific inhibitors demonstrating clinical activity in selected patients. These variant-specific differences highlight the functional heterogeneity of *KRAS*-driven signaling in CRLM (40). Persistent activation of the RAS/RAF/MEK/ERK pathway may lead to changes in cytokine release and reshapes myeloid cell behavior within the TME (41). *KRAS* mutations appear to tumor cells to secrete CSF-1, GM-CSF, and CCL2, which promotes the recruitment of hepatic macrophages and their polarization toward a M2-polarized TAMs phenotype, while also increasing the accumulation of myeloid-derived suppressor cells (MDSCs) within metastatic sites (28). In the context of *KRAS*-mutant CRLM, AKR1B10 is significantly elevated and contributes to neutrophil recruitment via the CXCL8/CXCR2 signaling axis, alongside enhanced glycolytic flux and lactate accumulation. The increased lactate subsequently promotes histone lactylation, thereby up-regulating PD-L1 expression and facilitating the transition of neutrophils toward an immunosuppressive N2 phenotype (42). At the same time, N2 neutrophils produce immunosuppressive cytokines, including IL-10 and TGF-β, which reinforce the suppressive TME (43, 44). Recent single-cell and functional studies further reveal the heterogeneity of tumor-associated neutrophils in CRC, demonstrating that oncogenic *KRAS* signaling can drive distinct neutrophil polarization states and reinforce immunosuppressive programs within the metastatic microenvironment (45).

### 2.3 Role of PI3K/AKT/mTOR signaling in CRLM

The PI3K/AKT/mTOR signaling pathway plays a crucial role in the metastatic progression of CRC. Aberrant activation of this pathway in CRC is commonly driven by upstream genetic alterations, including mutations in PIK3CA or loss of PTEN, leading to sustained activation of AKT and downstream mTOR signaling, which promotes tumor cell proliferation, survival, and metabolic reprogramming (46, 47). Exosomes derived from CRC promote changes in hepatic target cells by delivering specific miRNAs and other molecules, initiating a series of pro-metastatic responses. These exosome-mediated effects function as important modulators of the PI3K/AKT/mTOR pathway rather than its primary drivers (48).

Studies show that exosomes regulate M2 polarization of macrophages, enhance CXCL12/CXCR4 signaling, and activate the PI3K/AKT/mTOR pathway, thereby promoting the proliferation, invasion, and liver colonization of CRC tumor cells (49). In the early stages of liver metastasis, the PI3K/AKT/mTOR pathway not only activates hepatic stromal cells, particularly hepatic stellate cells (HSCs), but also promotes tumor cell proliferation, survival, and metastatic potential (50). During CRC metastasis, activation of the PI3K/AKT signaling pathway drives EMT by increasing mesenchymal marker expression and engaging EMT-specific transcriptional programs, thereby boosting the invasive and metastatic capabilities of tumor cells (51). Studies have demonstrated that serum exosome concentrations are markedly higher in patients with CRC and liver metastases than in healthy controls, suggesting a critical role for exosomes in tumor initiation and metastatic progression (52). However, whether serum exosome levels can reliably distinguish between localized and metastatic CRC remains incompletely defined, and further studies are needed to clarify their stage-specific clinical relevance. Research indicates that inhibition of the PI3K/AKT signaling pathway markedly suppresses CRC tumor cells (53).

## 3 Immunosuppressive microenvironment in CRLM

### 3.1 Immune suppressive functions of hepatic immune cells in CRLM

In CRC patients, CRLM is a major cause of death. As an immune-tolerant organ, the liver’s TME plays a crucial role in the progression of CRLM, filled with immune suppressive factors and immune cells that interact in complex ways to promote tumor immune escape. Specifically, innate immune cells in the liver, such as Kupffer cells (KCs), and stromal cells, like hepatic stellate cells (HSCs), interact with tumor cells and other immune cells to significantly enhance tumor growth and metastasis (54).

KCs are resident macrophages in the liver and play a crucial role in immune surveillance, antigen clearance, and immune tolerance, making them an essential component of the liver immune system (41). In the liver TME, KCs often exhibit immunosuppressive characteristics, primarily in the M2 (anti-inflammatory) state, which promotes immune evasion by tumors. The immune microenvironment of CRLM typically exhibits “cold” tumor features, where immunosuppressive cells, including Tregs and M2-polarized macrophages, produce cytokines such as IL-10 and TGF-β to inhibit local immune responses. The interaction between KCs and the TME contributes to the immune suppression that facilitates liver metastasis (55). By inducing *in situ* expansion and reprogramming of KCs, as demonstrated in mouse liver metastasis models, these cells can be transformed into the M1 phenotype, enabling direct tumor cell attack and the induction of inflammation to suppress tumor growth. Additionally, reprogrammed KCs can reduce the formation of an immunosuppressive microenvironment by clearing tumor-associated immunosuppressive factors, thereby restoring local immune activity (56).

HSCs are the most abundant non-parenchymal cells in the liver, making up 10% of all resident cells. When activated, they can affect the development of CRLM by remodeling the ECM (57). As showed in *in vitro* studies and preclinical models, activated HSCs interact with CRC cells through signaling axes to promote tumor growth and metastasis. Factors secreted by CRC cells, such as SDF-1, activate HSCs through the SDF-1/CXCR4 signaling pathway, enhancing their pro-tumor functions (58). Additionally, activated HSCs secrete ECM components, like collagen and fibronectin, altering the liver microenvironment and providing adhesion and colonization support for cancer cells, facilitating the formation of early tumor-pre-metastatic niches (59). Furthermore, HSCs release chemokines (such as CCL20) and inflammatory cytokines, modulating tumor cell behavior, attracting other immune and inflammatory cells to the liver, and creating an immunosuppressive environment that aids tumor growth and immune evasion (60).

**Figure 1:**
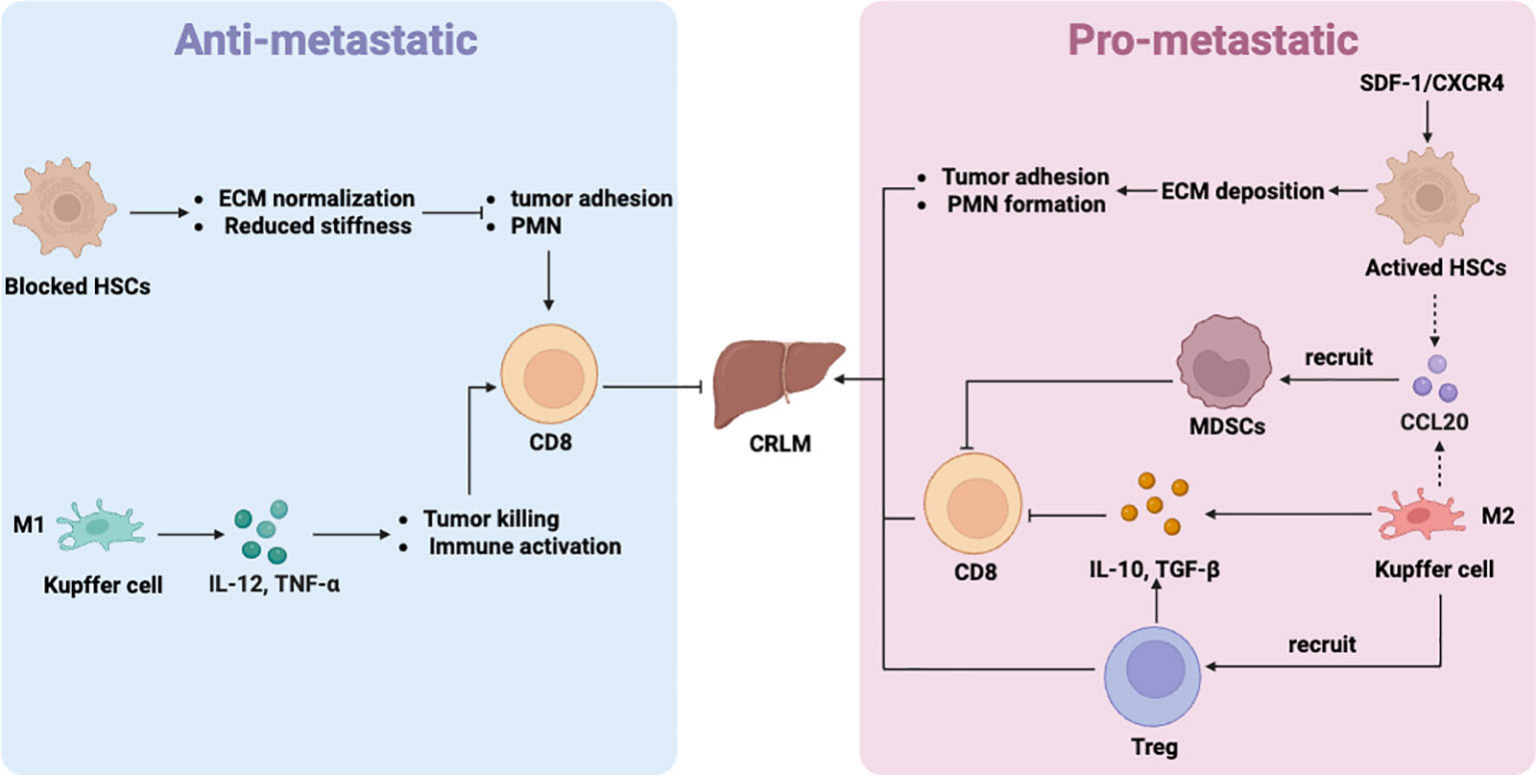
Dual roles of hepatic microenvironment remodeling in CRLM: KCs and HSCs. Pro-metastatic remodeling is characterized by M2-polarized KCs and Tregs releasing IL-10 and TGF-β, alongside activated HSCs driving ECM deposition through the SDF-1/CXCR4 axis, thereby supporting tumor cell seeding and immune suppression. In contrast, reprogramming KCs toward an M1 phenotype and limiting HSC activation can restore anti-tumor immunity, enhance CD8^+^T cell function, and disrupt the metastatic niche. The interplay between these opposing processes ultimately governs CRLM development. MDSCs, myeloid-derived suppressor cells; SDF-1, stromal cell-derived factor 1; CXCR4, C-X-C chemokine receptor type 4.

In CRLM, hepatic resident cells and stromal components cooperatively shape a permissive microenvironment for tumor progression. KCs often adopt an immunosuppressive phenotype, while HSCs-mediated ECM remodeling supports tumor cell seeding and expansion. Concurrently, the release of factors such as IL-10, TGF-β, and chemokines sustains an immunosuppressive milieu (20, 55). Owing to the liver’s intrinsic immune tolerance, CRLM typically displays an immune “cold” phenotype, marked by impaired effector T cell function and enrichment of suppressive immune populations. This microenvironmental context has been recognized as a major barrier to effective immune checkpoint blockade (ICB) (55). Moreover, hepatic and stromal elements further modulate immune cell recruitment and activity, promoting CD8^+^T cell dysfunction and the expansion of immunosuppressive subsets (**Figure 1**).

### 3.2 Regulation of CD8^+^T cell exhaustion in the CRLM microenvironment

Within the TME, CD8^+^T cells with dysfunctional phenotypes display reduced effector functions, including lower production of cytokines such as TNF, IL-2, and IFN-γ, as well as compromised proliferative capacity. Over time, these cells gradually progress toward an exhausted state, a condition that is strongly linked to unfavorable clinical outcomes and diminished responsiveness to immunotherapy (61). Studies have demonstrated that CD8^+^ tissue-resident memory T cells within CRLM share certain phenotypic features with conventionally exhausted T cells, including elevated expression of inhibitory markers such as PD-1 and CD39, as well as partial overlap in exhaustion-associated transcriptional programs (62). However, these cells are not fully functionally impaired. They retain proliferative activity, as indicated by Ki-67 expression, and preserve cytotoxic capacity marked by Granzyme B (GZMB) (63). In autologous Patient-Derived Xenograft models, they exhibit tumor-specific homing and persistence while maintaining a TRM phenotype. Collectively, these findings suggest that the hepatic metastatic niche may promote the development of a CD8^+^T cell state characterized by tissue residency combined with partial exhaustion, rather than driving a purely terminally exhausted phenotype (64).

Within the uniquely immune-tolerant environment of the liver, CD8^+^T cell function is readily constrained. Studies in mouse models have shown that the liver can selectively eliminate activated CD8^+^T cells, inducing apoptosis through mechanisms involving FasL signaling and nitric oxide (65). This process attenuates antitumor immune responses and creates conditions that facilitate the establishment of metastatic lesions (65). In CRLM, exhausted CD8^+^T cells within the TME are classified as M-type immune cells, based on single-cell transcriptomic analyses of clinical samples, with their phenotypic features primarily driven by tumor-intrinsic malignant programs rather than by the hepatic microenvironment itself. These cells are marked by high expression of LAYN, a transcriptomic marker of exhausted CD8^+^T cells, and enrichment of MKI67 proliferative CD8^+^T cell subsets, representing a tumor-driven exhaustion phenotype (66). In the immune microenvironment of CRLM, CD8^+^T cell exhaustion represents a dynamic continuum rather than a fixed state, progressing from progenitor exhausted cells (T_pex_) to terminally exhausted cells. Notably, T_pex_ cells retain proliferative capacity and responsiveness to ICB, whereas T_ex_ cells exhibit diminished effector function and limited reversibility, highlighting their distinct roles in immunotherapy response (67). T_pex_ cells retain partial proliferative capacity and self-renewal potential. However, under persistent immunosuppressive pressures within the liver–such as hypoxia, metabolic stress, and sustained inhibitory signaling from suppressive immune populations–their functional capacity gradually declines, ultimately leading to an irreversible terminal exhaustion phenotype (68). Moreover, the liver-specific metabolic milieu, together with inhibitory mediators derived from KCs and tumor cells, can reshape CD8^+^T cells at both epigenetic and transcriptional levels, thereby reinforcing the exhaustion program (69).Within the CRLM microenvironment, cancer-associated fibroblasts (CAFs) contribute to tumor progression in part through the secretion of MFAP2. Evidence suggests that MFAP2 activates the ITGB8–FAK–ERK1/2–ETS2–CYP27A1–LXRβ signaling cascade, thereby exerting dual effects on tumor growth and immune regulation (70, 71). This pathway not only promotes CRC cell proliferation, invasion, and hepatic colonization, but also restrains the activation and cytotoxic function of tumor-infiltrating CD8^+^T cells, ultimately reinforcing local immunosuppression (72). Evidence from clinical cohorts and multi-omics analyses indicates that a reduction in functional, non-exhausted CD8^+^T cells within metastatic lesions–or conversely, an expansion of exhausted subsets–is closely associated with increased risk of recurrence and poorer survival outcomes. These findings suggest that the proportion of terminally exhausted T cells may serve as a potential immunological marker for stratifying disease progression risk and prognosis in CRLM (73).

**Figure 2:**
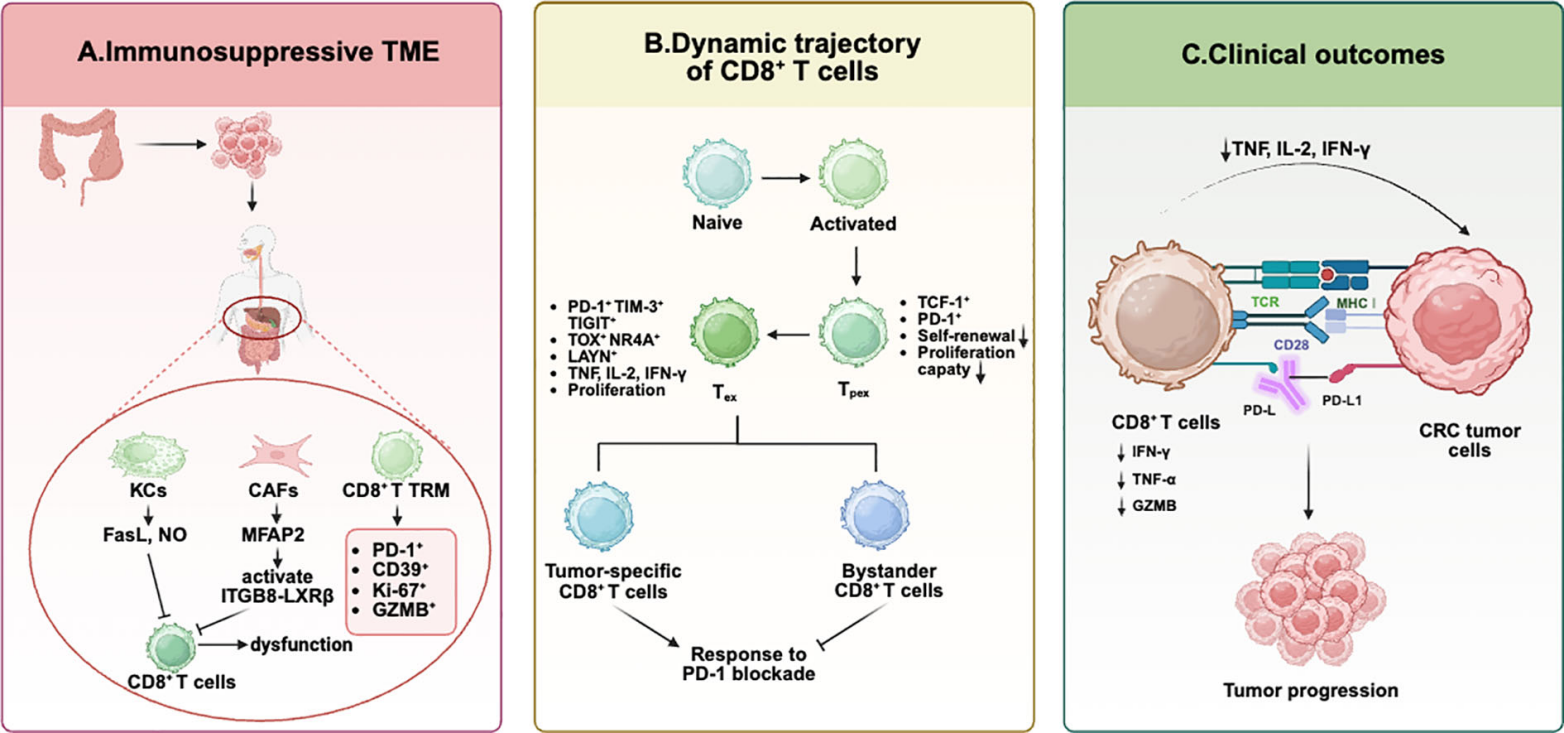
CD8^+^T cell exhaustion and immune evasion in the CRLM TME. **(A)** Immunosuppressive TME, where KCs and CAFs promote CD8^+^T cell dysfunction. **(B)** Dynamic trajectory of CD8^+^T cells from naïve to T_pex_ and T_ex_ states. **(C)** Clinical consequences of CD8^+^T cell exhaustion, including tumor progression and impaired response to ICB.

Emerging evidence indicates that exhausted CD8^+^T cells in CRLM can be broadly categorized into two functional subsets. One subset comprises tumor-specific CD8^+^T cells, which, under persistent tumor antigen stimulation, acquire classical exhaustion features characterized by upregulation of inhibitory receptors such as Programmed Death-1 (PD-1), TIM-3, and TIGIT, along with increased expression of transcription factors such as TOX and NR4A family members. Despite this exhausted phenotype, these cells often retain a TCF-1^+^ stem-like population, suggesting potential responsiveness to immunotherapeutic interventions (20). In contrast, bystander CD8^+^T cells are primarily driven by non-tumor antigens and exhibit a dysfunctional state. Although they may express certain exhaustion and inflammatory markers, their cytotoxic gene programs, including GZMB and IFN-γ, are markedly reduced. Moreover, the secretion of pro-inflammatory cytokines such as IL-17 may further reinforce the immunosuppressive microenvironment. These cells generally show limited benefit from PD-1 blockade (74, 75). In CRLM, PD-1 blockade alone is generally insufficient to rescue CD8^+^T cells that have progressed to a terminally exhausted state, particularly those co-expressing multiple inhibitory receptors. Moreover, most CRLM cases are microsatellite stable (MSS) and exhibit a relatively low neoantigen burden, resulting in a limited pool of tumor-specific CD8^+^T cells. In addition, a subset of infiltrating CD8^+^T cells represents dysfunctional bystander populations that are unlikely to be effectively reactivated by PD-1 inhibition. Collectively, these factors underlie the limited efficacy of PD-1 monotherapy (20, 76) (**Figure 2**).

### 3.3 Tregs-mediated immune suppression and T cell dysfunction in CRLM

Within the immunosuppressive TME of CRLM, Tregs accumulate prominently and function as key mediators of immune suppression, thereby contributing to tumor immune escape and the progression of metastatic disease. In both murine models and human CRLM tissues, the proportion of tumor-infiltrating Tregs is markedly higher in affected tissues compared with normal counterparts, indicating a significant enrichment of immunosuppressive cells within the TME (77). In CRLM, Tregs suppress antitumor immunity through multiple mechanisms. Notably, accumulating evidence in recent years indicates that Tregs in CRLM are not a homogeneous population, but exhibit pronounced functional heterogeneity, with distinct subsets exerting differential roles in immune regulation (78). By constitutively expressing elevated levels of CD25, Tregs competitively utilize IL-2, limiting both the activation and cytolytic function of effector T cells. In addition, by secreting immunosuppressive cytokines, including IL-10, IL-35, and TGF-β, Tregs inhibit CD8^+^ effector T cell activation and proliferation, diminish IFN-γ and TNF-α production, and thereby compromise antitumor immune responses (79, 80). In CRLM, IL-10 is predominantly produced by Tregs and M2-polarized TAMs. Further studies suggest that Tregs can be classified based on IL-10 expression. IL-10^+^ Tregs exhibit more stable regulatory functions and may be associated with favorable outcomes, whereas IL-10^-^ Tregs display a more pro-tumor immunosuppressive phenotype linked to effector T cell dysfunction and disease progression (81). This distinction underscores Tregs heterogeneity and supports more precise targeting strategies (82). Evidence indicates that blockade of IL-10 signaling can partially reverse CD8^+^T cell exhaustion. This effect is associated with decreased expression of inhibitory receptors such as PD-1 and TIM-3, restoration of the TCF-1^+^ stem-like CD8^+^T cell subset, and recovery of cytotoxic activity and cytokine production. Collectively, targeting IL-10 may help reprogram T cell function and enhance antitumor immune responses in CRLM (83). As observed in clinical samples and single-cell studies, Tregs differ markedly between primary hepatocellular carcinoma and CRLM. In CRLM, Tregs display a highly activated and strongly immunosuppressive phenotype, characterized by elevated expression levels of CTLA-4, CXCR4, PD-1, and ENTPD1 (CD39). In contrast, primary liver cancer is predominantly enriched in ICOS^+^ Tregs, while non-suppressive Tregs are less abundant and exhibit reduced Foxp3 demethylation and weaker inhibitory effects on effector T cell proliferation (78).

Partial hepatectomy in mice may promote CRLM by inducing an inflammatory premetastatic niche and reshaping the hepatic immune landscape. These changes suggest a shift toward an immunosuppressive microenvironment. Notably, pHx elevates IL-6, TGF-β, and VEGF levels and enhances ECM remodeling, all of which are closely linked to the recruitment, differentiation, and maintenance of Tregs (84). On this basis, Tregs accumulate in liver metastatic lesions and contribute to local immune tolerance. In addition to classical immunosuppressive mechanisms, Tregs also rely on metabolic adaptation to sustain their function (81). In addition, metabolic factors play a critical role in maintaining Tregs stability and suppressive function (84, 85). *In vitro* studies, lactate accumulation and hypoxic conditions support Foxp3 expression and enhance Tregs adaptation to the TME, while fatty acid oxidation and mitochondrial metabolism enable sustained suppressive activity under nutrient-limited conditions (86). Meanwhile, as suggested by *in vitro* studies and preclinical tumor models, the tryptophan–kynurenine metabolic pathway contributes to immunosuppression by promoting Tregs differentiation and expansion while suppressing effector T cell responses (87). In parallel, CD39/CD73-mediated adenosine production inhibits CD8^+^T cell activation and cytotoxic function, thereby establishing a metabolically driven immunosuppressive network (88). In *in vitro* studies and preclinical models, tumor-infiltrating Tregs can utilize lactate as an energy source and maintain stable Foxp3 expression under hypoxic and nutrient-limited conditions. Enhanced fatty acid oxidation and mitochondrial metabolism further support their suppressive activity, leading to persistent inhibition of effector T cell responses and promoting tumor immune evasion (89). Therapeutic approaches targeting Tregs are shifting from simple depletion toward functional reprogramming. Strategies such as IL-10 signaling blockade and combination with immune checkpoint inhibitors have emerged as promising avenues, offering potential targets for improving immunotherapy in CRLM (83, 90).

### 3.4 TAMs-driven immunosuppressive remodeling and metastatic progression in CRLM

Within the TME of CRLM, M2-polarized TAMs represent another key immunoregulatory population that cooperate with Tregs to establish an immunosuppressive network (34). Recent studies have highlighted the pronounced heterogeneity of M2-polarized TAMs in CRLM. Immunosuppressive subsets, particularly SPP1^+^ and MRC1^+^ populations, are predominant and engage in intricate spatial interactions with tumor cells and stromal components (54).

Notably, advances in single-cell sequencing and spatial transcriptomics have substantially refined our understanding of M2-polarized TAM heterogeneity in CRLM in recent years. Emerging evidence indicates that M2-polarized TAMs in CRLM can be stratified into distinct functional subsets (91). Among them, SPP1^+^ M2-polarized TAMs represent one of the most prominent pro-metastatic populations, showing marked enrichment within liver metastatic lesions and contributing to tumor progression through the promotion of angiogenesis, ECM remodeling, and immunosuppressive signaling (54). In addition, EEF1G^+^ macrophages have been identified as a metastasis-associated subset, closely linked to EMT, immunometabolism, reprogramming and unfavorable clinical outcomes (92). Furthermore, under conditions such as chemotherapy-associated steatohepatitis, MARCO^+^ macrophages have been observed to expand significantly and are associated with enhanced CD8^+^T cell exhaustion as well as increased risk of metastatic recurrence (93). More recently, integrative analyses combining single-cell and spatial transcriptomics have identified additional subsets, including HLA-DRB5^+^ macrophages, which appear to modulate T and NK cell functions through ligand–receptor interaction networks (94).

Within the CRLM microenvironment, M2-polarized TAMs release fibrinogen-like protein 2 (FGL2) via exosomes, thereby supporting the preservation of tumor cell stemness and triggering the generation of neutrophil extracellular traps, fostering an immunosuppressive state. Meanwhile, FGL2 also enhances the recruitment and immunosuppressive function of Tregs, collectively accelerating liver metastatic progression (95, 96). SPP1^+^ macrophages recruit Tregs to hepatic metastatic lesions of CRC by secreting chemokines such as CCL22 and CCL17. In parallel, by producing TGF-β and IL-10, these cells promote the conversion of CD4^+^T cells into Foxp3^+^ Tregs, thereby contributing to an immunosuppressive microenvironment (97). Tumor-associated neutrophils promote the infiltration of M2-type macrophages and Tregs through the production of CCL2 and CCL17. In turn, the accumulated M2 macrophages and Tregs further reinforce the immunosuppressive TME within the liver, thereby facilitating tumor progression and metastatic dissemination (80).

During tumor development in mouse CRLM models and *in vitro* studies, enhanced glycolytic activity leads to lactate accumulation within the TME. Elevated intratumoral lactate levels subsequently promote the differentiation of M2-polarized macrophages and Tregs (98). Under these conditions, CD8^+^ T cells exhibit a pronounced exhausted phenotype, characterized by impaired activation and proliferative capacity, along with a progressive decline in effector function and cytotoxicity. In contrast, CD4^+^T cells showed a decrease in the number of cells, impaired Th1-related immune activity, and abnormally enhanced immunosuppressive function of Tregs (99). Lactic acid accumulation promotes increased infiltration of Tregs in the microenvironment of CRLM tumors. At the same time, M2-polarized TAMs further enhance the expansion and function of Tregs by secreting immunoregulating cytokines, regulating tumor cell migration and phenotyping, thus enhancing the immunosuppressive environment (100).

In CRLM, PD-1 signaling pathway is not only limited to the local TME, but also related to systemic immunosuppression through its impact on peripheral immunoregulation. Circulatory PD-1 refers to the overall existence of PD-1 in peripheral blood, including soluble PD-1and circulating immune cells expressing PD-1, which are mainly expressed in PD-1^+^T cells (101). TAMs can sustain activation of the SPHK1-PD-L1 axis, thereby amplifying PD-1 signaling (102). This process facilitates the induction and recruitment of Tregs, enhances their immunosuppressive capacity, and ultimately promotes their accumulation within CRLM lesions (103).

## 4 Current emerging therapeutic strategies for CRLM

### 4.1 Chemotherapy combined with targeted therapy

Once CRC progresses to liver metastasis, overall prognosis is generally unfavorable. Patient survival is influenced by a range of clinical and pathological variables. In addition to histological subtype, factors such as tumor differentiation, depth of invasion, extent of metastatic spread, and biological aggressiveness are closely associated with disease progression and long-term outcomes (104). Collectively, these characteristics contribute to variability in treatment response and disease progression (105). The primary treatment approaches for CRLM include surgical resection and systemic chemotherapy. However, upon diagnosis, only approximately 10-20% of patients are considered eligible for potentially curative surgery (106). Therefore, to improve the prognosis and survival outcomes of patients with CRLM, combining conventional treatments with emerging targeted therapies or immunotherapeutic approaches has been explored as a novel therapeutic strategy (2). In first-line treatment for metastatic CRC, bevacizumab and the anti-EGFR monoclonal antibodies cetuximab and panitumumab are commonly administered in combination with standard chemotherapy regimens. Many of the clinical trials evaluating these strategies have included a substantial proportion of patients with CRLM. Notably, cetuximab and panitumumab exert their antitumor effects by blocking EGFR signaling, whereas bevacizumab primarily targets tumor angiogenesis (107).

EGFR overexpression is observed in approximately 60%-80% of CRC cases, making it a relevant molecular target for therapeutic intervention. Cetuximab, a chimeric monoclonal antibody, selectively binds to EGFR and competes with endogenous ligands such as EGF, thereby preventing receptor activation and downstream signaling. Clinical evidence indicates that, when combined with standard chemotherapy regimens, cetuximab can help delay tumor progression and reduce the risk of disease worsening (108). Anti-EGFR antibodies such as panitumumab have been shown to improve objective response rates and increase the likelihood of conversion to resectable disease in patients with *RAS* wild-type tumors (109). However, for individuals with liver-limited, initially unresectable *RAS* wild-type CRC, the optimal multidisciplinary treatment approach remains unsettled (110). Although combining chemotherapy with targeted agents such as anti-VEGF or anti-EGFR antibodies has been associated with improved tumor shrinkage and increased resection rates, treatment efficacy appears to vary according to primary tumor location. Nevertheless, tumor reduction may still be achieved in selected cases, potentially facilitating subsequent local treatment. Therefore, the use of anti-EGFR antibodies in right-sided *RAS* wild-type CRLM should be considered on an individualized basis (106, 111, 112). In patients with CRLM harboring *RAS* or *BRAF* mutations, the efficacy of anti-EGFR agents such as cetuximab and panitumumab is substantially reduced, limiting their clinical benefit. For this molecular subgroup, treatment strategies more commonly favor bevacizumab combined with chemotherapy, with the goal of achieving improved disease control and potentially extending survival (113). In patients with *RAS*-mutant metastatic CRC, multiple randomized clinical trials, including the CRYSTAL study, have consistently demonstrated that the addition of cetuximab provides no improvement in OS, progression-free survival (PFS), or response rate. Furthermore, pooled analyses have confirmed that patients with BRAF-mutant tumors derive limited benefit from anti-EGFR therapy, highlighting its restricted clinical utility in this molecular subgroup (114).

Bevacizumab, the first clinically approved anti-angiogenic agent, exerts its therapeutic effect by selectively binding to various isoforms of vascular endothelial growth factor A (VEGF-A), thereby preventing its interaction with vascular endothelial growth factor (VEGF) receptors and suppressing downstream signaling activation (115). Given the central role of the VEGF pathway in tumor angiogenesis, inhibition of this pathway reduces tumor vascularization, thereby restricting tumor growth and metastatic dissemination. Consequently, bevacizumab is primarily utilized in malignancies that are highly dependent on angiogenesis for progression (116). Bevacizumab in combination with chemotherapy represents a key therapeutic strategy for improving outcomes in patients with CRLM. However, despite its clinical benefits, the development of acquired resistance remains a common challenge, ultimately limiting long-term treatment efficacy. Studies indicate that anti-VEGF therapy can drive structural remodeling of the TME. Excessive deposition of ECM components leads to the formation of a dense stromal network, which restricts the penetration of chemotherapeutic agents into liver metastatic lesions and disrupts normal tumor vascular architecture, thereby compromising drug delivery (117, 118). In parallel, increased matrix stiffness activates the FAK/YAP signaling cascade, stimulating lipolysis in hepatic stellate cells. The released fatty acids are subsequently utilized by tumor cells to enhance fatty acid oxidation, promoting metabolic adaptation. This shift in metabolic programming ultimately strengthens tumor cell tolerance to chemotherapy and diminishes the overall efficacy of combination treatment strategies (119). Emerging evidence indicates that anti-VEGF monotherapy is often inadequate for sustained disease control in CRLM, largely due to stromal remodeling and metabolic adaptation driving therapeutic resistance. Therefore, combinational strategies targeting the tumor stroma and metabolic pathways, together with immunotherapy and multimodal interventions, are increasingly being investigated to improve clinical outcomes (**Table 1**).

**Table 1:** Clinical integration of monoclonal antibodies in the systemic management of CRLM.

Monoclonal antibody names	Target	Combined chemotherapy regimen	Clinical application scenarios	Key indicated population
Bevacizumab (22, 23, 115, 120)	VEGF-A	FOLFOX, FOLFOXIRI, FOLFIRI	first-line treatment, conversion therapy, high tumor burden	preferred therapy for patients with *RAS*/*BRAF* mutations and right-sided primary tumors
Cetuximab (109, 110, 121)	EGFR	FOLFIRI, FOLFOX	first-line treatment, conversion treatment	*RAS* wild-type, left-sided primary tumors**;** limited or no benefit in right-sided tumors
Panitumumab (110, 122)	EGFR	FOLFOX	first-line treatment	*RAS* wild-type, left-sided primary tumors; caution in right-sided tumors
Anti-EGFR + BRAF inhibitor (123, 124)	EGFR + BRAF	targeted combination + chemotherapy	later-line or selected first-line strategies in BRAF-mutant disease	BRAF V600E-mutant CRC; not recommended as monotherapy, requires combination strategy

### 4.2 Locoregional interventional strategies for CRLM

With the continuous advancement of therapeutic strategies for CRLM, locoregional interventional therapies have gradually become an integral component of the treatment framework. For patients with unresectable disease or those considered potentially resectable after treatment, these approaches provide important options for tumor control and for achieving conversion to surgical resection (125).

#### 4.2.1 Radiofrequency ablation

Radiofrequency ablation (RFA) is widely used as a local minimally invasive treatment for patients with CRLM who cannot undergo surgical resection, and it may also serve as a complementary approach to liver surgery. By delivering heat through an electrode, RFA raises the temperature of the surrounding tissue, leading to protein denaturation within cells and ultimately causing coagulative necrosis and tumor cell destruction (126). The combination of RFA with chemotherapy and targeted therapy has been shown to improve both median PFS and OS in patients with unresectable CRLM (127). RFA is a well-established technique with a favorable safety profile and is particularly suitable for patients with solitary or limited liver metastases measuring ≤3 cm in diameter (126).

#### 4.2.2 Microwave ablation

Although RFA has become a well-established technique in clinical practice, it still has certain limitations, including a relatively small ablation zone, slower heating efficiency, and susceptibility to the “heat sink effect” when lesions are located near large blood vessels, which may result in incomplete ablation (128). For intermediate-sized CRLM measuring 3-5 cm, microwave ablation (MWA) can still achieve favorable local tumor control. However, reported local control rates vary widely from 22% to 90%, likely due to differences in study design and tumor characteristics (129, 130). Compared with RFA, the therapeutic efficacy of MWA is less susceptible to the “heat-sink effect” caused by adjacent blood flow, which makes it more suitable for treating liver metastases located near large vessels. Importantly, repeatability should be considered a general feature of locoregional therapies, including MWA, RFA, and other modalities, allowing retreatment in cases of tumor recurrence and contributing to sustained local control and long-term disease management (131). A recent bibliometric study focusing on research trends in the local management of CRLM indicates that the combination of local ablation and immunotherapy has gained growing attention in recent years. Accumulating evidence suggests that integrating ablative techniques with immune-based strategies may improve therapeutic outcomes and help overcome some limitations of single-modality treatment (132).

#### 4.2.3 Transarterial chemoembolization

Transarterial chemoembolization (TACE) has gradually evolved from a purely palliative approach into an integral component of multidisciplinary treatment strategies for CRLM (133). This shift is largely attributed to its ability to exploit the predominant arterial blood supply of metastatic lesions in the liver (134). Through selective catheterization, chemotherapeutic agents can be directly delivered into tumor-feeding arteries, followed by embolic materials that obstruct vascular perfusion. This dual mechanism achieves a synergistic effect by combining high local drug concentrations with ischemia-induced tumor necrosis (132, 135). In recent years, drug-eluting bead TACE (DEB-TACE) has further refined this approach by enabling controlled and sustained drug release, thereby enhancing intratumoral drug retention while minimizing systemic exposure (136). Emerging clinical evidence suggests that, in patients with unresectable disease or resistance to standard systemic therapies, TACE may PFS and reduce tumor burden in a subset of cases, potentially facilitating subsequent conversion to surgical resection (137).

#### 4.2.4 Hepatic arterial infusion chemotherapy

Because CRLM are predominantly supplied by the hepatic artery, whereas approximately 70% of the blood flow to normal liver parenchyma originates from the portal vein, hepatic arterial infusion chemotherapy (HAIC) enables direct drug delivery through the hepatic artery. This approach allows higher drug concentrations to accumulate within tumor lesions while minimizing systemic exposure (138). On this basis, HAIC combined with targeted therapy has emerged as an effective interventional option for patients with unresectable colorectal CRLM who have failed standard systemic chemotherapy (139). This liver-directed strategy, centered on arterial drug infusion, can achieve improved intrahepatic tumor control while maintaining relatively low systemic toxicity. The therapeutic benefit appears more pronounced in patients without extensive extrahepatic disease, making it a potential approach for both conversion therapy and palliative management (26). Hepatic arterial infusion pump (HAIP)–based locoregional chemotherapy combined with IL-12-targeted immunotherapy represents a strategy that integrates local tumor control with immune modulation. By taking advantage of the ability of HAIP to deliver chemotherapy directly to hepatic lesions, this approach enables precise eradication of CRLM tumor cells while simultaneously improving the TME through targeted delivery of IL-12. In doing so, it enhances endogenous antitumor immune responses and helps overcome limitations associated with conventional treatments (140). Consequently, growing research efforts have begun to focus on immunotherapy-based approaches, particularly strategies aimed at modulating the immunosuppressive TME to improve therapeutic responses in patients with CRLM.

### 4.3 Immunotherapeutic approaches for CRLM

In recent years, immunotherapy has emerged as a major research focus in the treatment of CRC. Among these approaches, ICIs have demonstrated notable clinical benefits in a subset of patients. MWA-based combination immunotherapy has emerged as a potential therapeutic approach for CRLM. Beyond inducing local tumor destruction, MWA can trigger the release of tumor antigens and promote immune activation. IL-21 enhances this effect by boosting the cytotoxic capacity of CD8^+^T cells and NK cells, whereas PD-1 inhibitors reinvigorate dysfunctional T cells. The integration of these strategies may augment local immune responses and generate systemic antitumor effects, thereby contributing to the control of disseminated liver metastases and offering new possibilities for multimodal CRLM management (141). Evidence suggests that microwave ablation (MWA) leads to increased expression of LAG3 in tumor-infiltrating lymphocytes (TILs). When LAG3 blockade is combined with MWA, the ablation-induced immune response is amplified, resulting in enhanced proliferation and cytotoxic function of CD8^+^ TILs. This strategy has been shown to delay tumor growth and extend survival in the MC38 tumor model (55). Although preclinical studies have suggested that combining MWA with ICIs may enhance antitumor immune responses, current clinical evidence in CRLM remains limited. Emerging immune checkpoints such as TIGIT and TIM-3 are increasingly recognized as important regulators of the tumor microenvironment. In the MC38 tumor model, the combination of MWA and TIGIT blockade significantly increased the infiltration of TILs particularly CD8^+^ effector T cells, suggesting a potential immunomodulatory effect of this strategy (142, 143). However, the clinical efficacy of MWA combined with ICIs requires further validation in prospective studies. Blocking TIGIT signaling restored CD8^+^T cell activity and enhanced the production of cytotoxic molecules such as IFN-γ and PRF1, thereby strengthening antitumor immune responses (142).

**Figure 3:**
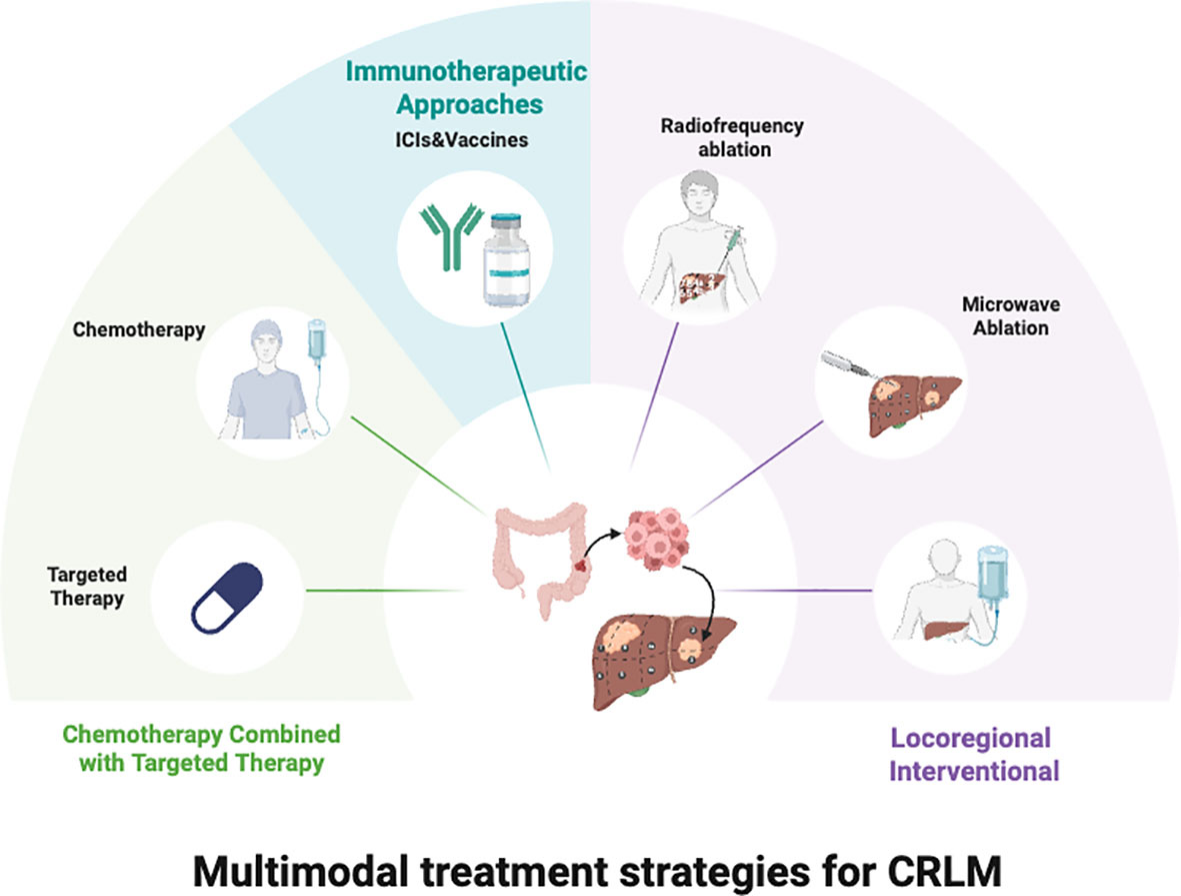
Multimodal treatment strategies for CRLM. The diagram illustrates the main therapeutic approaches for CRLM, categorized into three major arms: systemic therapies (including chemotherapy, targeted therapy, combined chemo-targeted therapy, and immunotherapeutic strategies such as ICIs and vaccines), and locoregional interventional therapies (including radiofrequency ablation, microwave ablation, and other local ablative procedures).

ICB using pembrolizumab monotherapy has demonstrated improved clinical outcomes in patients with microsatellite instability–high/deficient mismatch repair (MSI-H/dMMR) metastatic CRC (144). In contrast, MSS/pMMR tumors, which represent the majority of CRLM cases, generally show limited responsiveness to PD-1 blockade (21). Furthermore, the presence of liver metastases has been associated with reduced efficacy of immunotherapy, highlighting the immunosuppressive nature of the hepatic microenvironment (145). In MSI-H/dMMR metastatic CRC, PD-1/PD-L1 checkpoint blockade has demonstrated notable clinical benefit, with improved response rates and survival outcomes. As a result, these agents have been increasingly adopted as part of the therapeutic approach for CRLM, supported by pivotal randomized clinical trial evidence demonstrating significant survival benefit in this patient population (144, 146, 147). In patients with MSI-H/dMMR CRLM, PD-1 inhibitors such as nivolumab and pembrolizumab have demonstrated favorable therapeutic efficacy, improving objective response rates and PFS. However, patients with microsatellite stable/proficient mismatch repair (MSS/pMMR) CRLM generally show limited responses to PD-1 inhibitor monotherapy, although low-frequency objective responses have been reported in selected patients (148, 149). In CRLM immunotherapy, nivolumab combined with the CTLA-4 inhibitor ipilimumab can enhance immune activation and improve control of liver metastases compared with monotherapy, although it may increase the risk of immune-related adverse events. In addition, the exploratory combination of the PD-L1 inhibitor botensilimab and the CTLA-4 inhibitor balstilimab has demonstrated measurable objective response and disease control rates in early-phase clinical studies, although current evidence remains preliminary, in patients with advanced MSI-H/dMMR CRLM, providing a potential option for those with limited benefit from single-agent immunotherapy (150–152). Evidence indicates that tumor mutational burden (TMB) thresholds can help predict treatment response and disease progression in patients receiving ICIs. Lower TMB levels are generally associated with earlier disease progression, while patients with higher TMB are more likely to benefit from combination therapy targeting CTLA-4 and PD-1 (153). With the identification of emerging immune checkpoint targets, TIGIT has gained increasing research interest. Studies suggest that the combination of the TIGIT inhibitor tiragolumab and the PD-L1 inhibitor atezolizumab may produce synergistic immune activation in CRC, partially restoring T cell function in MSS tumors (154). In addition, ongoing clinical studies, such as the PURPLE trial, are evaluating this combination as a neoadjuvant strategy for CRLM, highlighting its potential in expanding immunotherapeutic options (155). Preclinical studies suggest that simultaneous blockade of LAG-3 and TIM-3 can alleviate T cell exhaustion and enhance antitumor immune responses, showing inhibitory effects on tumor growth and intrahepatic metastasis in models of CRLM (156). However, although much of the early evidence originated from preclinical and early-phase clinical studies, recent phase III trials have demonstrated measurable clinical activity in this setting. In MSS/pMMR CRLM, which typically exhibits a low tumor mutational burden, the efficacy of ICB alone remains limited, although objective responses have been observed in a subset of patients (24, 157). Therefore, larger clinical studies are needed to further evaluate the long-term efficacy, safety, and optimal treatment strategies of this approach. In addition, identifying predictive biomarkers–such as LAG-3 and TIM-3 expression levels–may help enable more precise selection of patients who are most likely to benefit (148). CRLM immunotherapeutic targets modulate the tumor microenvironment and enhance clinical outcomes (**Table 2**). The major therapeutic strategies for CRLM are summarized (**Figure 3**).

**Table 2:** Emerging immunotherapeutic targets and agents in CRLM.

Targets	Representative drug	Level of evidence	MSI/MMR relevance	Mechanism	Key reference
LAG-3	Relatlimab, Eftilagimod alpha	Phase I/II or preclinical, depending on agent	All-comers	Reverse CD8^+^ T cell exhaustion and reduce Treg-mediated immunosuppression	(158)
TIM-3	Sabatolimab	Phase I	All-comers	Inhibit CD8^+^ T cell exhaustion, restore effector T cell function, and reduce immunosuppressive cell activity	(159)
TIGIT	Tiragolumab	Phase I/ongoing CRLM trial	MSS/pMMR	Restore CD8^+^ T cell and NK cell function while reducing Treg-mediated immunosuppression	(160)
CXCL12–CXCR4	Plerixafor	Preclinical/translational	Not MSI-specific	Blocking tumor cell homing and immune exclusion mechanisms	(161)
CXCR3	CXCR3 agonist	Preclinical	Not MSI-specific	Enhancing NK cell infiltration and cytotoxic antitumor activity	(162)
LDHA	LDHA inhibitors	Preclinical	Not MSI-specific	Targeting immunometabolism and reprogramming	(163)
TGF-β	Galunisertib	Preclinical/Phase II in rectal cancer, not CRLM-specific	MSS/pMMR	Targeting immunosuppressive cell function	(164)
FAK	Defactinib	Preclinical	Not MSI-specific	Targeting ECM remodeling to overcome immune barriers	(165)
*KRAS*	mRNA-5671/V941 vaccine	Phase I/exploratory	Mutation-specific	Targeting *KRAS* mutation–derived neoantigens	(163)

## 5 Future perspectives and challenges

Most patients with CRLM belong to the MSS subtype and generally exhibit limited responses to ICIs. Compared with MSI-H tumors, MSS tumors are typically characterized by reduced immune cell infiltration together with increased accumulation of TAMs, reflecting a typical “cold” tumor immune phenotype. In addition, abnormalities in antigen presentation machinery, aberrant activation of β-catenin signaling, and enhanced TGF-β pathway activity may further impair the efficacy of ICIs by strengthening Treg–mediated immunosuppression and suppressing NK cell function (55, 166). The therapeutic efficacy of immunotherapy in CRC is largely determined by microsatellite instability status, commonly defined as MSI-H/dMMR versus MSS/pMMR. Tumors with dMMR or MSI-H usually display a high tumor mutational burden and generate abundant neoantigens, thereby enhancing tumor immunogenicity and improving sensitivity to ICIs. In contrast, the majority of metastatic CRC cases are classified as pMMR/MSS tumors, which generally exhibit lower immunogenicity. Moreover, accumulating evidence indicates that the presence of liver metastases itself may further attenuate the effectiveness of immunotherapy regardless of MSI status (21). However, this effect appears to be context-dependent and varies according to MSI/MMR status and treatment strategy. In MSS/pMMR metastatic CRC, this negative impact is particularly pronounced and consistently associated with reduced response rates and shorter progression-free survival, whereas in MSI-H/dMMR disease, the detrimental effect appears less absolute and may be regimen-dependent, with emerging evidence suggesting that combination immunotherapy may partially mitigate this effect (147). The liver possesses a unique immune-tolerant microenvironment that can suppress systemic antitumor immune responses, which represents an important factor contributing to resistance to immunotherapy. Future studies are needed to further elucidate the underlying mechanisms of immune resistance and to develop more effective combination therapeutic strategies.

In this context, accurately identifying patients who are most likely to benefit from immunotherapy has become an important research focus. Currently, commonly used predictive biomarkers for immunotherapy include MSI/dMMR, PD-L1 expression, and TMB (167). However, the clinical utility of these indicators remains limited due to several challenges, such as the lack of standardized detection methods and substantial heterogeneity across tumor regions and among individual patients. Therefore, the identification of more reliable biomarkers with greater predictive value remains a key direction for future research, including TILs, immune-related gene signatures, and circulating immune biomarkers (168).

However, the clinical benefits of immunotherapy alone remain limited in a proportion of patients, and single-modality treatment often fails to achieve satisfactory outcomes. Consequently, increasing attention has been directed toward combining immunotherapy with other therapeutic approaches, including chemotherapy, anti-angiogenic therapy, and local treatments such as ablation or radiotherapy (22, 125, 151). These combination strategies may enhance antitumor immunity through multiple mechanisms, including promoting tumor antigen release, improving immune cell infiltration within the TME, and further stimulating T cell activation and effector function. Among these approaches, the integration of radiotherapy or local ablation with immune checkpoint inhibitors has been considered a promising therapeutic strategy, and related studies are continuing to expand in this area (169).

## 6 Conclusion

CRLM is a major determinant of clinical outcomes in patients with CRC. Its development and progression are closely associated with the formation of an immunosuppressive TME and aberrant activation of multiple signaling pathways. In recent years, advances in systemic chemotherapy, locoregional interventions, and ICIs have provided new therapeutic opportunities for CRLM. Nevertheless, treatment efficacy remains limited due to immune tolerance within the hepatic microenvironment and the emergence of therapeutic resistance. Therefore, further investigation of the underlying immunoregulatory mechanisms and optimization of combination treatment strategies are required to improve therapeutic outcomes and patient prognosis.
